# Vascular density in age-related macular degeneration after one year of antiVEGF treatment with treat-and-extend and fixed regimens

**DOI:** 10.1371/journal.pone.0229388

**Published:** 2020-02-26

**Authors:** Miklós D. Resch, Anikó Balogh, Gábor Gy Deák, Zoltán Z. Nagy, András Papp

**Affiliations:** 1 Department of Ophthalmology, Semmelweis University, Budapest, Hungary; 2 Department of Ophthalmology, Uzsoki Hospital, Budapest, Hungary; 3 Department of Ophthalmology and Optometry, Medical University of Vienna, Vienna, Austria; University of Pecs, HUNGARY

## Abstract

Treatment of neovascular age-related macular degeneration (nAMD) with VEGF can be performed with several posologies. The purpose of our cross-sectional study was to analyze retinal vessel density by quantitative OCT-angiography (OCT-A) and to compare treat-and-extend (T&E) and fixed treatment protocols to a control group with dry AMD. Altogether 48 patients were enrolled: 13 eyes with T&E protocol ranibizumab treatment (group A) and 17 eyes with fixed regimen aflibercept therapy (group B), the control group comprised 18 eyes with dry AMD (group C). One year after the start of the treatment, quantitative OCT-A (AngioVue—Optovue, Fermont, USA) was performed: superficial and deep retinal vessel densities were analyzed in the foveal and parafoveal regions. Our results show, that the density of retinal superficial vasculature in the fovea was not different between the treatment groups (A: 25.9±9.1%; B: 24.3%±8.9), neither from group C (25.6±4.8%). Superficial parafoveal vascular density of the retina, however, was decreased in both treated groups (A: 46.7±9.1%, B: 42.9±6.1%, C: 49.7±4.9%). In the deep retinal plexus, vascular density was lower in both treatment groups compared to that of in controls in both the foveal and parafoveal area (A: 29.8±6.3%, B: 32.5±6.9%, C: 36.4±1.7% and A: 46.3±3.8%, B: 47.1±5.3%, C: 49.7±4.9%, foveal and parafoveal respectively). Our data suggest, that after one year of anti-VEGF treatment, reduced macular vessel density in three of the four examined vascular regions can be found independent of the treatment regimen.

## Introduction

Neovascular age-related macular degeneration (nAMD) despite the effective antiangiogenic treatment is still one of the leading causes of visual impairment in industrialized countries [1]. Antiangiogenic (antivascular endothelial growth factor–antiVEGF) therapy achieved effective control of choroidal neovascularization (CNV). Randomized controlled trials have shown that different treatment protocols can be recommended for the best anatomic and functional results in nAMD [2]. The protocols include fixed (monthly or bimonthly) injections, pro re nata (PRN), treat-and-extend (T&E) [3, 4]. The latest guidelines recommend T&E and fixed protocols for the optimal treatment of nAMD with ranibizumab and aflibercept [5, 6].

The traditional gold standard of CNV detection is fluorescein and indocyanine green angiographies aided by OCT. Since the introduction of optical coherence tomography-based angiography (OCT-A), which is a non-invasive method to image retinal and choroidal circulation, the diagnosis of CNVs shifted more to a multimodal imaging approach [7]. OCT-A provides biomarkers to identify the activity of the disease and its prognostic values as well [8, 9]. With the aid of OCT-A, the morphologic changes and quantitative vascular parameters of the CNV in nAMD have been evaluated. It has been shown, that choroidal vascular density significantly decreases after antiVEGF injections [8], and signs of vascular abnormalization with antiangiogenic therapy for choroidal neovascularization can be seen [9]. The reduction of the size of CNV is in correlation with the beneficial visual effects of antiVEGF treatment. Cennamo et al have demonstrated with OCT-A, that after the loading dose of three bevacizumab injections, retinal vascular density does not change significantly. Frequently, however, after a long-term antiangiogenic treatment significant atrophy of the neuroretina, retinal pigment epithelium (RPE), and choroid is observed, which is in correlation with the visual impairment in long-term treated nAMD [10]. You et al described the reduced retinal vessel density in the geographic atrophic form of dry AMD [11], and Sorour found a significant decrease of macular vascular density in diabetic retinopathy treated with antiVEGF [12].

To our knowledge, no quantitative OCT-A findings are available to evaluate the long-term effect of antiVEGF therapy on retinal vessel density in nAMD. Our hypothesis is that the retinal circulation might be affected by nAMD and by the antiangiogenic treatment. This study aimed to analyse retinal vessel density by quantitative OCT-A in patients, treated for one year with different treatment regimens for nAMD, compared with an age-matched control group of eyes with dry AMD.

## Materials and methods

### Study design

In a prospective cross-sectional institutional study, a total of 48 eyes of 48 subjects (18 male and 30 female) were recruited from the retina outpatient clinic of the Department of Ophthalmology at Semmelweis University. The study followed the tenets of the Declaration of Helsinki; applicable national and local requirements; and was approved by the Regional and Institutional Committee of Science and Research Ethics. All patients signed written informed consent before entering the study.

### Participants

Consecutive patients with nAMD were divided into 2 groups according to the intravitreal antiVEGF treatment, performed as on the drug label. The treatment was initiated 12 months before the study, a therapeutic agent was determined and prescribed as part of routine clinical care. Both treatment types were reimbursed by the national healthcare system, patients were randomly selected for the study.

Group A consisted of 13 eyes of 13 patients, who received intravitreal 0.5mg in 0.05ml of ranibizumab (Lucentis, Novartis, Switzerland) treatment, following a T&E protocol (https://www.medicines.org.uk/emc/product/307/smpc). The T&E protocol was applied, according to the EuRetina Guidelines, i.e., patients received a repeated intravitreal injection at each visit but the time interval to the next visit was extended stepwise by 2 weeks, in case of no disease activity (based on visual acuity, OCT and fundus image) [13]. If disease activity recurred, the treatment interval was shortened, accordingly.

Group B contained 17 eyes of 17 patients, who were treated with intravitreal 2 mg in 0.05ml of aflibercept (Eylea, Bayer, Germany). Aflibercept posology followed the fixed protocol: 3 initial monthly loading dose, and later fixed bimonthly injections up to 12 months. The therapeutic protocol was followed according to the valid official Summary of Product Characteristics (SmPC) of the drug. (https://www.medicines.org.uk/emc/product/2879/smpc).

Control Group. A group of controls was enrolled from healthy volunteers or patients without macular pathology besides dry AMD (AREDS Stage 1 and 2). The presence of geographic atrophy was exclusive.

Inclusion criteria were originally treatment-naive patients with available clinical data, one year (50–54 weeks) after treatment initiation with an antiVEGF drug. Patients fulfilling the above criteria were scheduled for a study visit, including visual acuity, biomicroscopic, and OCT-A examinations. Only patients with clear ocular media were included, in order to get proper quality images. CNV was classified retrospectively, according to the FA and OCT images, taken before starting the treatment. Only eyes with type 1 and type 2 CNVs were included in the study. Polypoidal choroidal vasculopathy and Type 3 CNV were exclusion criteria. Eyes with geographic atrophy of the macula revealed by fundoscopy and OCT were excluded from the study.

Further ocular exclusion criteria were: Myopia or hyperopia over 6 Diopters; opaque media, limiting good image quality (only records with scan quality 6/10 and above were included). Uncontrolled glaucoma, previous vitreoretinal surgery or macular pathology, other than nAMD were exclusive.

Non-ocular exclusion criteria were any type of diabetes, uncontrolled hypertension, and systemic use of antiangiogenic drugs.

### Examinations, image acquisition, and analysis

All subjects underwent a comprehensive ophthalmic examination, including best-corrected visual acuity (VA) assessment (ETDRS Charts), slit lamp, and fundus examinations. After pupil dilation, OCT-A was performed, using the AngioVue OCT-A system (RTVue-XR Avanti, OptoVue, Fremont, CA, USA). Scans with the highest resolution were obtained in the central 3×3 mm area, centered on the foveola. The superficial capillary plexus was detected automatically between the internal limiting membrane (ILM) and the inner plexiform layer (IPL); and the deep capillary plexus between the IPL and the outer plexiform layer (OPL). Segmentation errors were manually controlled for each layer to avoid the potential artifacts due to the distortion of anatomy with nAMD.

Foveal thickness was measured in the central 1.0 mm area. Superficial and deep vessel density (VD) was evaluated in the whole image, in the central 1 mm area (fovea), and in the 3 mm ring-shaped parafoveal area. Superficial non-flow area and foveal avascular zone (FAZ) areas were measured, using the built-in AngioAnalytics software (Version ReVue 2018.0.0.18) OptoVue system with an automated segmentation algorithm.

### Statistical analysis

Group A, B, and the control groups were compared with Mann-Whitney and Fisher-exact tests with the Statistica 13.4 software (Tibco Software Inc). The Spearman rank correlation test was performed to investigate the role of age, VA, or the number of injections. Subgroup analysis with the Mann-Whitney test was performed for the comparison of CNV lesion types.

## Results

Patients’ characteristics are summarized in **Table 1**. There was no difference in age and gender in Group A and B from the control group. Visual acuity was better in the control group than in Groups A and B, according to the disease under treatment. The number of injections, the type of CNV, and the follow-up time was not different in Group A and B.

**Table 1:** Demographic and clinical data of patients under different antiVEGF therapy and the control group. Data are shown as mean (SD).

	Group A	Group B	Control
**N**	13	17	18
**Male/female**	3/10	7/10	8/10
**Age (years)**	76.8 (9.4)	75.4 (9.2)	73.3 (6.9)
**VA (ETDRS letters)**	65.1 (5.1)	61.2 (6.0)	77.1 (6.1)*
**CNV Type (1/2)**	10/3	12/5	N/A
**No. Of IVI**	7.1 (1.7)	6.7 (0.5)	N/A
**Follow-up (weeks)**	51.7 (2.3)	52.3 (3.1)	N/A

Group A. ranibizumab (Lucentis) T&E treatment group.

Group B. aflibercept (Eylea) fixed protocol treatment group.

IVI- intravitreal injections.

*p<0.05 (Mann-Whitney and Fisher-exact test).

Quantitative OCT-A results are summarized in **Table 2**. No significant difference was found in scan quality among the groups. Central retinal thickness was higher in Group A and B, compared to control but it was not different between Group A and B.

**Table 2:** Results of OCT-A examinations. Superficial and deep vascular density in the fovea, parafovea and in the whole image. Non-flow area and foveal avascular zone (FAZ). Data are shown as mean (SD).

	Group A	Group B	Control	p (Group A vs Control)	p (Group B vs Control)	p (Group A vs B)
**Scan quality**	7.1 (0.9)	7.2 (1.0)	7.8 (0.8)	0.07	0.06	0.95
**Foveal thickness (μm)**	287.4 (54.1)	282.1 (61.3)	257.3 (34.4)	0.05*	0.04*	0.45
**Superficial fovea (%)**	25.9 (9.1)	24.3 (8.9)	25.6 (4.8)	0.92	0.58	0.62
**Superficial parafovea (%)**	46.7 (2.8)	42.9 (6.1)	49.7 (5.0)	0.04*	0.001*	0.03*
**Superficial whole image (%)**	36.4 (5.3)	36.9 (6.1)	45.1 (3.4)	<0.001*	<0.001*	0.80
**Deep fovea (%)**	29.8 (6.3)	32.4 (6.9)	36.2 (1.7)	0.003*	0.04*	0.28
**Deep parafovea (%)**	46.3 (3.7)	47.1 (5.3)	49.9 (0.7)	0.004*	0.04*	0.62
**Deep whole image (%)**	43.4 (3.8)	45.2 (4.9)	47.3 (1.2)	0.003*	0.01*	0.25
**Superfical non-flow area (mm^2^)**	0.62 (0.38)	0.44 (0.12)	0.32 (0.10)	0.01*	0.003*	0.12
**FAZ area (mm^2^)**	0.44 (0.21)	0.28 (0.08)	0.22 (0.05)	0.002*	0.01*	0.02*

Group A. ranibizumab (Lucentis) T&E treatment group.

Group B. aflibercept (Eylea) fixed protocol treatment group.

*p<0.05 (Mann-Whitney test).

**Fig 1:**
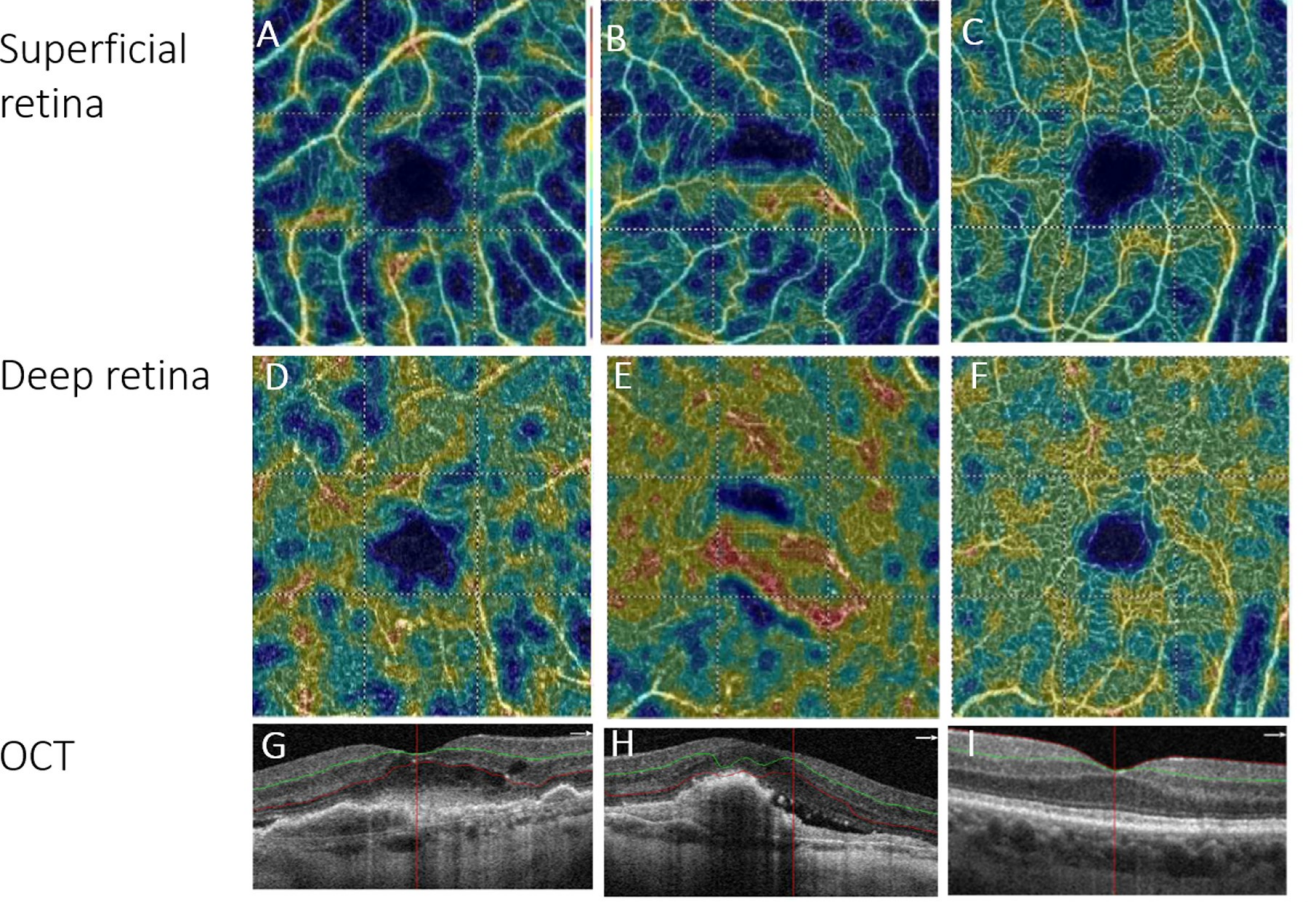
OCT-A imaging of the superficial (A-C) and deep (D-F) retinal capillary plexus, using the automated AngioAnalytics software in Group A (first column), B (second column) and control group (third column). Note the lower density in treated eyes, compared to control eyes.

**Fig 2:**
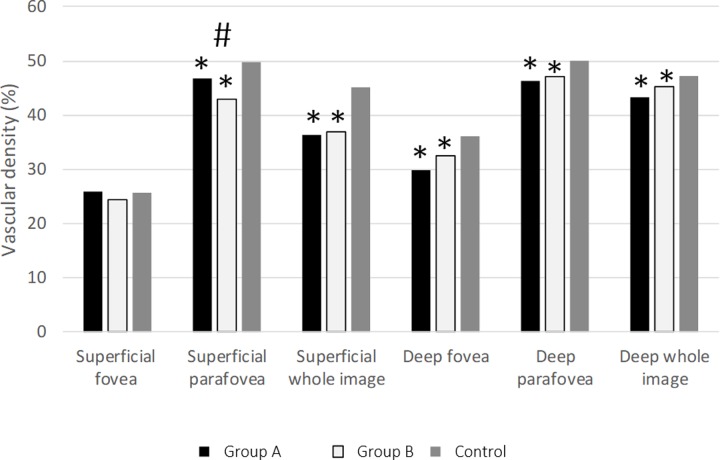
Diagram of the mean of superficial and deep retinal vascular density in all groups. *p<0.05 from the control group, †p<0.05 between Group A and B.

Superficial retinal vessel density. The density of superficial retinal vasculature in the fovea was not different between the treatment groups (group A: 25.9 ± 9.1%; group B: 24.3% ± 8.9), neither from the control group (25.6 ± 4.8%) (**Fig 1**). Parafoveal vascular density was lower in the treated groups (46.7 ± 9.1%, 42.9 ± 6.1%, 49.7 ± 4.9%, group A, group B, and control, respectively) (**Fig 2**).

Deep retinal vascular density in the fovea was lower in both treatment groups, compared to control (group A: 29.8 ± 6.3%, group B: 32.5 ± 6.9%, control: 36.4 ± 1.7%); and parafoveally as well (46.3 ± 3.8%, 47.1 ± 5.3%, 49.7 ± 4.9%, group A, group B, and control, respectively).

**Fig 3:**
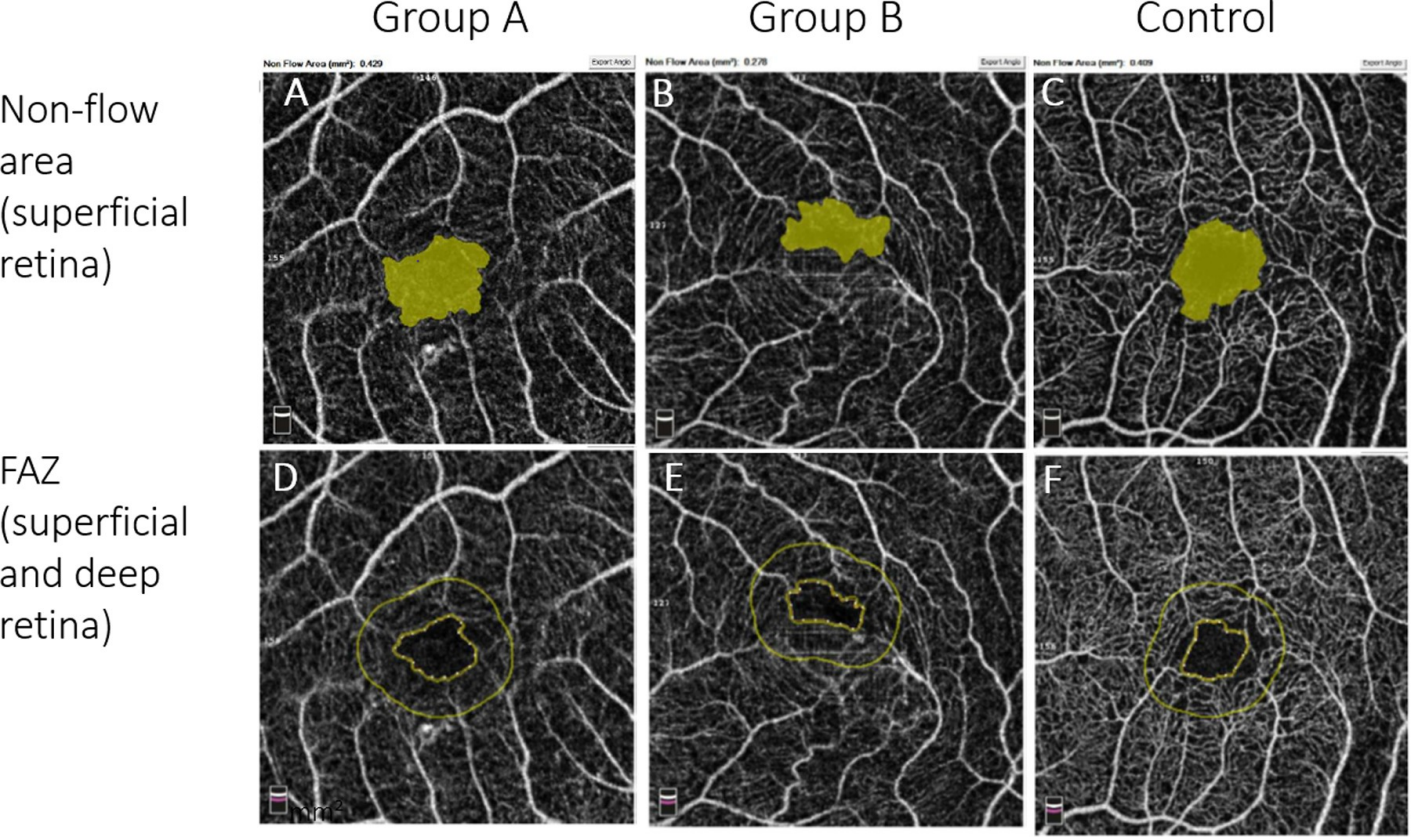
OCT-A imaging of the foveal avascular zone (A-C, FAZ), and the non-flow area (D-F). The areas are calculated by the automated AngioAnalytics software. Note the greater FAZ and non-flow areas in Group A (first column), B (second column) compared to the control group (third column).

**Fig 4:**
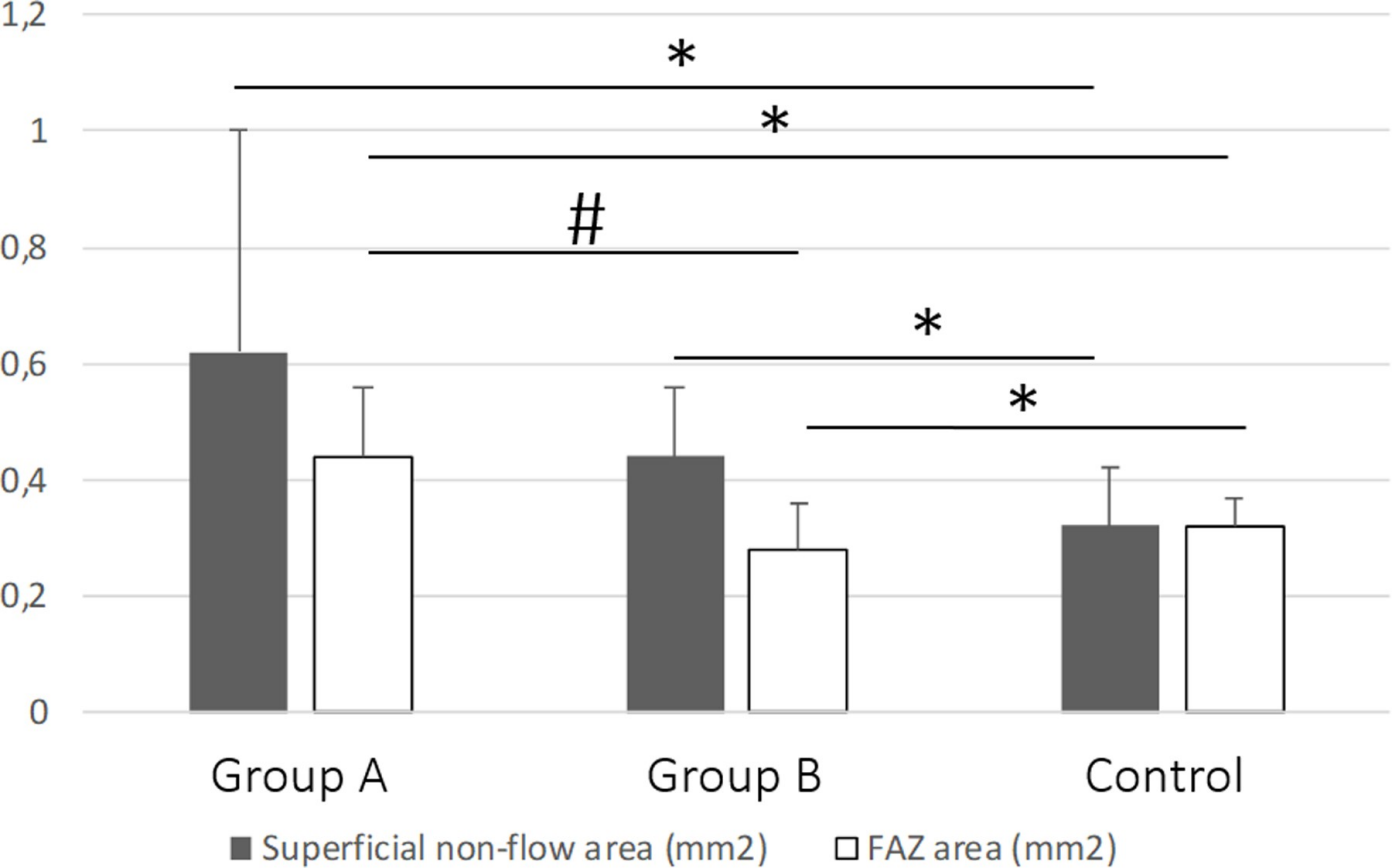
Diagram of the mean and SD of superficial non-flow area and FAZ. *p<0.05 from the control group, †p<0.05 between Group A and B.

Non-flow and FAZ areas in both AMD groups were higher than those of the control group (**Fig 3)**. Considering OCT-A measurements, there was no significant difference in the mean non-flow and FAZ area between Groups A and B (**Fig 4)**.

None of the OCT-A parameters showed a significant correlation with age, VA or the number of injections. CNV-type influenced neither vascular density nor non-flow area and FAZ.

## Discussion

The treatment-protocol, used with the different antiVEGF agents for nAMD has been changing ever since their introduction into the ophthalmological armamentarium. Multiple randomized clinical trials have demonstrated the beneficial effects of antiVEGF therapy; however, there is growing evidence that simultaneous atrophic changes in the neuroretina and retinal pigment epithelium develop during antiVEGF therapy [14]. VA and central retinal thickness are the key variables to assess the efficacy of treatment, which is highly influenced by the treatment protocol used (i.e., the number of injections) in the first two years [15]. Updated treatment protocols are aiming to administer neither undertreatment nor overtreatment of the patients. The real-life, long-term followup study by Munk et al [16] found macular atrophy only in 73.5% of subjects, where patients received less yearly injections than patients in clinical trials. One of the factors of atrophy can be the decrease of retinal circulation in the macula, which can be seen by angiography.

There is growing knowledge on how antiVEGF therapy affects choroidal neovascularisations in patients with neovascular AMD, but there is much less information on how antiVEGF therapy affects the retinal circulation [9]. To our knowledge our study is the first one to compare the quantitative measurement of the retinal vessels, using OCT-A, in connection antiVEGF therapy to control choroidal neovascularization in patients treated with different antiVEGF agents and different treatment protocols. One year after treatment initiation, decreased retinal vascular density was seen in patients treated with both examined antiVEGF agents compared to age-matched controls. In the foveal area, the superficial vascular density was retained, but it decreased in the parafovea; and the density of the deep retinal vessels in the fovea and the parafovea was lower than in the controls.

There was no difference between T&E and fixed protocols in terms of relevance, in a 1-year perspective. In our study, we have shown that in the treated nAMD, the non-flow area and FAZ increase in the retina, irrespectively of the treatment protocol. The results fit in the literature data since ranibizumab T&E and aflibercept fixed protocol administration are considered equal in clinical studies and real-life as well [17]. Since we used a T&E protocol with ranibizumab, the number of injection, given do not differ in the two groups. A significant difference would have occurred if we had treated the patients according to the protocols in VIEW 1 and 2 studies (i.e., monthly fixed ranibizumab) [18].

Interestingly, we found no correlation of retinal vessel density with age and visual acuity in our cohort. The type of CNV is a factor that determines the natural progression of the disease even with PDT therapy (MARINA, ANCHOR), but if patients receive antiVEGF treatment, VA gain is quite similar in cases of Type 1 and 2 CNVs [19, 20]. In the MARINA trial, minimally classic and occult lesions were treated; in the ANCHOR trial mainly classic type CNV-s, which showed slightly better results in BCVA improvement. One of the limitations of our study is that we excluded PCV and RAP lesions, thus we have no information about those CNV types. Matsumoto evaluated T&E and fixed protocols of aflibercept treatment, in all types of CNV-s and found it to be equally effective. Our concept of excluding PCV and RAP was to have more easily comparable groups. A further limitation of the study is that we lack information on the baseline vascular density data of the eyes, before starting the treatment. Other studies show that if baseline BCVA, CRT, and lesion types are similar, vascular density is expected to be similar as well [21]. The first versions of Angioplex software had segmentation difficulties in the case of the irregular macula (e.g. high neuroretinal detachment due to nAMD). At the time of the treatment start of our patients, the new software was not available yet.

OCT-A has been found to be a reproducible method to measure retinal vessel density with the automated AngioAnalytics software [22]. OCT-A has been shown to be a valuable tool in the follow up of CNV, after antiVEGF treatment in patients with nAMD but there are only a few reports on the retinal vascular system of nAMD patients [9]. Faatz et al. [23] evaluated the activity of the CNV: measured the total vessel length, the number of segments. They calculated the fractal dimension of the CNV before and after therapy. Commercially available software; however, are not yet suitable for the automated detection of these parameters.

Rispoli et al. [24] examined eyes in the first month after a single antiVEGF injection, due to nAMD. They demonstrated that OCT-A is also suitable to highlight quantitative choriocapillaris vascular density changes (fluctuation) around neovascularization (dark halo).

McClintic et al. [25] followed up patients on PRN antiVEGF therapy with quantitative OCT-A, measured CNV vessel area and membrane area, which reduced after three months, as compared to baseline. No evaluation of retinal vessels was performed in their pilot study. Miere et al. [26] compared PRN vs. monthly loading-dose regimen antiVEGF therapy with OCT-A: the CNV size-quantitative analysis showed a significant decrease in the lesion area with monthly injections. However, no significant change in the lesion area was observed during PRN antiVEGF therapy.

Pilotto et al [27] investigated the short-term (48 hours) changes of type 1 CNV, secondary nAMD after antiVEGF treatment. Quantitative and qualitative vascular and morphological macular changes revealed that the CNV mean area, the neuroretinal and the retinal pigment epithelial detachment and the cystoid edema significantly reduced after treatment. Fine CNV vessel density decreased by 75% but larger CNV vessel density remained stable at 66.7%.

Hikichi et al investigated nAMD patients in a treatment-free period after long-term antiVEGF treatment and found that Choroidal neovascularization enlargement and features may guide treatment timing in eyes with exudative-free periods [28].

In accordance with Cennamo’s findings [9], we can state that antiVEGF therapy can be so effective in successful in the treatment of nAMD, because it significantly decreases the permeability of choroidal neovascular vessels selectively without decreasing the blood flow in the retinal and choroidal circulations. Our findings, however, point out, that after long-term treatment, the retinal vessel density still can be affected by repeated antiVEGF treatment.

In conclusion, our results suggest that patients with nAMD, receiving antiVEGF therapy, due to active CNV, have lower retinal vascular density than age-matched control subjects, one year after therapy initiation. The agent used to treat and the regimen used for treatment did not result in a difference in vascular density. To our best knowledge, this study is the first to quantitatively compare superficial and deep macular vascular density after different treatment protocols. Further studies are necessary to examine the effect of vascular density decrease on visual function, and clinical course of patients, to determine, whether vascular density measurements could be used as a surrogate measure to predict clinical outcomes. Our study demonstrated that OCT-A provides exact data on the decreased vascular density of the macula, after the antiVEGF treatment. Prospective studies are required to highlight the fine changes in retinal vascular changes exactly.

## Supporting information

S1 Table The original dataset used for calculations. (PDF)
