# Supplementary material for: Vascular density in age-related macular degeneration after one year of antiVEGF treatment with treat-and-extend and fixed regimens
Source: PLoS One. 2020 Feb 26;15(2):e0229388. doi: 10.1371/journal.pone.0229388 (PMC7043775; doi:10.1371/journal.pone.0229388)
Supplement: S1 Table — (PDF) [file pone.0229388.s001.pdf]

Pt No.	Age	Eye	VA	CNV	Scan qual	Drug	No. treat	SPF fovea	SPF parafovea	SPF whole	D fovea	D parafovea	D whole	SPF non-flow	FAZ
1	77	OD	0,60	1,00	6	L	5	28,6	50,7	48,5	30,1	50,1	48,3	0,417	0,340
2	77	OS	0,05	1,00	7	L	9	16,7	47,1	35,2	24,1	48,3	47,1	0,709	0,560
3	66	OS	0,40	2,00	8	L	8	27,2	45,3	34,6	30,4	49,2	47,2	0,372	0,290
4	83	OD	0,80	2,00	6	L	7	27,8	47,9	31,6	31,5	50,2	46,3	0,310	0,270
5	82	OS	0,40	2,00	7	L	7	23,0	46,1	34,7	33,0	45,3	42,3	0,526	0,430
6	91	OD	0,60	1,00	6	L	6	32,5	41,1	29,5	36,2	44,1	40,1	0,263	0,198
7	74	OD	0,80	1,00	9	L	11	39,4	46,8	30,5	35,4	49,1	46,2	0,208	0,190
8	63	OD	0,20	1,00	7	L	8	39,7	49,3	31,9	40,1	44,1	42,1	0,671	0,492
9	85	OD	0,30	1,00	8	L	5	9,0	50,3	39,5	17,2	40,2	37,4	1,107	0,560
10	90	OD	0,30	1,00	7	L	6	16,1	43,6	36,5	23,6	39,1	37,3	1,490	0,850
11	77	OD	0,40	1,00	6	L	7	18,2	48,3	39,4	22,9	44,2	40,2	0,984	0,760
12	70	OD	0,50	2,00	8	L	8	27,2	43,3	41,6	29,5	49,4	46,7	0,599	0,430
13	63	OS	0,20	2,00	8	L	6	31,9	47,4	40,1	33,1	48,5	42,5	0,370	0,290
**Mean**	**76,8**		**0,43**		**7,15**		**7,15**	**25,92**	**46,70**	**36,43**	**29,78**	**46,29**	**43,36**	**0,617**	**0,435**
**SD**	**9,4**		**0,23**		**0,99**		**1,68**	**9,12**	**2,80**	**5,32**	**6,33**	**3,75**	**3,84**	**0,38**	**0,21**
14	72	OD	0,30	2,00	6	E	6	28,3	50,1	32,4	30,1	46,0	44,1	0,321	0,270
15	74	OS	0,16	1,00	6	E	7	41,6	39,8	43,1	29,5	42,1	41,0	0,473	0,320
16	74	OD	0,50	2,00	7	E	6	21,0	45,4	25,7	27,5	42,4	40,9	0,456	0,350
17	83	OD	0,40	2,00	7	E	7	28,7	48,1	32,1	30,2	45,2	43,1	0,350	0,270
18	91	OD	0,60	2,00	6	E	7	28,0	49,5	33,5	34,5	42,5	40,1	0,330	0,290
19	85	OD	0,25	1,00	8	E	6	43,1	47,7	44,7	44,2	46,1	45,4	0,241	0,190
20	83	OS	0,06	1,00	7	E	7	27,9	39,0	29,9	29,6	39,1	38,1	0,469	0,290
21	66	OD	0,30	2,00	8	E	7	14,8	35,4	33,3	26,6	47,7	45,3	0,559	0,206
22	70	OD	0,20	1,00	6	E	6	10,3	50,1	45,8	23,6	54,7	51,5	0,715	0,486
23	68	OD?	0,50	2,00	7	E	7	23,3	45,3	41,1	28,1	40,8	39,2	0,424	0,339
24	72	OD	0,40	1,00	9	E	7	21,1	39,7	38,0	39,6	45,8	45,3	0,477	0,220
25	62	OS	0,50	2,00	7	E	6	17,1	43,4	39,6	33,8	50,8	47,9	0,478	0,292
26	79	OS	0,30	2,00	8	E	7	30,9	34,8	34,9	48,6	54,3	52,5	0,278	0,148
27	84	OD	0,60	1,00	9	E	7	18,0	30,3	30,2	37,8	58,0	55,5	0,522	0,207
28	64	OS	0,50	2,00	7	E	7	18,1	48,2	44,0	28,3	49,4	47,3	0,482	0,339
29	90	OS	0,20	1,00	8	E	7	15,7	37,0	36,4	23,5	45,0	42,7	0,573	0,285
30	65	OD	0,4	2	6	E	7	24,8	45,0	43,6	36,1	50,8	48,9	0,309	0,235
**Mean**	**75,4**		**0,36**		**7,18**		**6,71**	**24,28**	**42,87**	**36,96**	**32,45**	**47,10**	**45,22**	**0,439**	**0,279**
**SD**	**9,25**		**0,16**		**1,01**		**0,47**	**8,91**	**6,08**	**6,08**	**6,98**	**5,28**	**4,90**	**0,12**	**0,08**
31	65	OS	0,6	0	8	0	0	17,6	38,0	37,8	36,3	49,9	49,3	0,4	0,2
32	75	OS	0,7	0	8	0	0	22,4	47,6	46,0	36,9	49,0	48,7	0,4	0,2
33	67	OS	0,7	0	9	0	0	19,8	43,1	39,5	36,2	49,7	48,1	0,4	0,2
34	78	OD	0,8	0	8	0	0	29,3	45,5	42,3	35,4	50,1	48,1	0,2	0,2
35	78	OS	0,8	0	9	0	0	25,9	51,6	44,1	33,2	49,6	49,1	0,3	0,2
36	81	OD	1	0	8	0	0	32,5	50,4	45,6	38,0	50,0	48,2	0,2	0,2
37	81	OS	1	0	7	0	0	22,6	47,4	44,1	35,9	48,3	46,7	0,2	0,2
38	65	OD	1	0	8	0	0	23,8	56,0	50,2	39,2	50,3	47,2	0,4	0,2
39	65	OS	0,6	0	6	0	0	20,8	47,5	44,7	37,3	51,0	48,0	0,5	0,2
40	67	OD	0,8	0	7	0	0	32,8	60,1	52,1	38,1	50,1	47,3	0,2	0,2
41	67	OS	1	0	8	0	0	32,1	54,5	48,1	35,1	49,9	45,2	0,2	0,2
42	83	OD	1	0	9	0	0	25,2	48,2	43,2	36,2	50,1	47,2	0,2	0,2
43	73	OD	0,8	0	7	0	0	20,6	52,3	47,8	33,4	51,3	46,5	0,4	0,4
44	77	OD	1	0	7	0	0	23,8	49,2	46,3	34,6	49,3	45,2	0,4	0,3
45	76	OD	1	0	8	0	0	31,4	53,0	46,7	39,1	49,9	46,3	0,3	0,3
46	65	OD	1	0	8	0	0	29,2	52,8	45,2	35,2	49,7	47,2	0,3	0,2
47	72	OD	1	0	7	0	0	29,7	47,9	43,3	37,1	50,1	46,3	0,2	0,2
48	85	OD	1	0	8	0	0	22,5	48,8	45,2	35,2	50,2	46,9	0,4	0,3
**mean**	**73,3**		**0,88**		**7,78**		**0,00**	**25,65**	**49,66**	**45,12**	**36,24**	**49,92**	**47,31**	**0,318**	**0,221**
SD	6,92		0,15		0,81		0,00	4,85	4,97	3,41	1,72	0,66	1,18	0,10	0,05
